# Investigation of the *in vivo* and *in silico* wound healing property of *Retama raetam* (Forssk) Webb & Berthel. leaves extract

**DOI:** 10.3389/fphar.2025.1621579

**Published:** 2025-07-31

**Authors:** Hafsia Bouzenna, Hmed Ben Nasr, Najla Hfaeidh, Ahlem Missaoui, Angelo Maria Giuffrè, Karim Hosni, Samira Jebahi, Naceur Mejri

**Affiliations:** ^1^ Laboratory of Biotechnology and Biomonitoring of the Environment and Oasis Ecosystems (LBBEEO), Faculty of Sciences of Gafsa, University of Gafsa, Gafsa, Tunisia; ^2^ Department of Life Sciences, Faculty of Sciences of Gafsa, University of Gafsa, Gafsa, Tunisia; ^3^ Department of AGRARIA, University Mediterranea of Reggio Calabria, Reggio Calabria, Italy; ^4^ Laboratory of Natural Substances, National Institute of Research and Physico and Chemical Analysis, Sidi Thabet, Tunisia; ^5^ Laboratory of Energy and Matter Research, National Center of Nuclear Science and Technology, Sidi Thabet, Tunisia; ^6^ Laboratory of nuclear biotechnology and technology, National Center of Nuclear Science and Technology, Sidi Thabet, Tunisia

**Keywords:** *Retama raetam* (Forssk) Webb & Berthel, wound healing, bioactive phenolics, antioxidant, antimicrobial, anti-inflammation, molecular docking

## Abstract

In this study, the wound healing properties of the leaf aqueous extract of *Retama raetam* (Forssk) Webb & Berthel was investigated *in vivo* and *in silico*. The HPLC-DAD profiling of bioactive compounds allowed the identification of five phenolics including quercetin, kaempferol, naringenin, myricetin and caffeic acid. The aqueous extract has been found to significantly inhibit microbial growth in the wounded tissue contributing thereby to its cleansing. The topical application of the aqueous extract accelerated wound closure and enhanced the re-epithelialization and restoration of damaged wound skin. The wound healing activity of the *R. raetam* which was supported by histopathological observations exceeded that of the standard wound healing cream biafine. Concomitantly, the wound-repairing action was associated with a reduced oxidative stress as revealed by the decrease of lipid peroxidation versus the activation of antioxidant enzymes SOD, CAT and GPx. *In silico* study showed that naringenin and to a less extent myricetin have the highest bending capacity to the active site of pro-inflammatory cytokines TNF-α and IL-1β receptors. These results indicate that *R. raetam* could be considered as a consolidated source of putative bioactive phenolics with excellent antioxidant, antimicrobial, anti-inflammatory and wound healing properties, offering thereby experimental and theoretical supports for its application in the treatment of burn skin wounds.

## 1 Introduction

Burn wounds consist of dermal and epidermal injuries that are characterized by disturbed structural and functional skin integrity. Skin wounds complications include bacterial infections, systemic inflammation, and delayed tissue regeneration (Wang, 2018). In the inflammatory phase during the early stage of wound healing, macrophages and monocytes are intensively recruited in response to overexpression of proinflammatory cytokines (IL-8, IL-6, IL-1β, and TNF-α) and angiogenic factors. This initial response is crucial in cleaning up tissues and expelling pathogens, and contaminants preparing tissue for the healing process (Burgess et al., 2022). In the subsequent proliferative phase of wound healing, the activation of keratinocytes and fibroblasts by cytokines (IL-6) and growth factors (e.g., transforming growth factor TGF-α, epithelial growth factor EGF, fibroblast growth factor, GM-CSF) contributes to the recovery of damaged extracellular matrix (ECM) via production of collagen, and restored the vascular networks (Gurtner et al., 2008). In the last phase called remodeling phase or maturation, new epithelium and scar tissue with a highly organized collagen matrix and the maximum tensile length are formed (Zulkefli et al., 2023).

The complex healing process is often accompanied with hypermetabolism-related oxidative stress owing to increased rates of gluconeogenesis, glycogenolysis, protein synthesis, lipogenesis, gluconeogenesis and hormone production (Soriano and Stanford, 2023). Despite their key roles in the wound healing process (e.g., leucocytes recruitment, activation of monocytes and macrophages, and their corresponding inflammasome, activation of platelets and neovascularization, formation of ECM, cleansing wound tissues, activation of cell signaling pathways and re-epithelialization, etc.), the overproduction of reactive oxygen species (ROS) and reactive nitrogen species (RNS) could negatively impact this healing process leading to prolonged inflammation and ultimately chronic wounds (Hunt et al., 2024).

To reestablish cellular redox homeostasis, exogenous supplementation with antioxidant-rich extracts/molecules could represent excellent therapeutic strategies targeting the prevention of oxidative stress and/or enhancing the endogenous antioxidant system (Wang et al., 2023). To ensure a balanced state of oxidative stress, several antioxidants including vitamins E and C, nicotinamide, carotenoids, phenolic compounds, ubiquinones, uric acid, and glutathione, among others have been proposed and/or used (Schäfer and Werner, 2008). Antioxidant-rich extracts from different plant species such as *Albizia julibrissin*, *Arnebia euchroma*, *Betula pendula, Betula pubescens*, *Centella asiatica*, *Hippophaë rhamnoides, Juglans regia*, *Lawsonia inermis*, and mixtures of *Matricaria chamomilla* and *Rosa canina* are known for their effectiveness in the regeneration and wound healing of burned tissue (Skowrońska and Bazylko, 2023).

*Retama raetam* (Forssk) Webb & Berthel is a medicinal shrub of the Fabaceae family, native to North Africa, Eastern Mediterranean countries and Middle East (Mittler et al., 2001). In folk medicine, *R. raetam* is used in the treatment of hypertension, diabetes, rheumatism, skin diseases, fever, and inflammation (Tahraoui et al., 2007; León-González et al., 2018). It has also demonstrated antioxidant, antimicrobial, anti-inflammatory, diuretic, hepatoprotective and nephroprotective effects (Omara et al., 2009; Algandaby, 2015). These biological properties are attributed to flavonoids (saponarin, retamacins, apigenine, quercetin, kaempferol, orientin, and their derivatives), vitamins (e.g.*,* ascorbic acid), carotenoids, organic acids (e.g., malic acid, piscidic acid), fatty acids (e.g., linoleic and linolenic acids), volatile oils (e.g., α-humulene, nonanal, linalool) and alkaloids (e.g., retamine, cytisine, lupinine, sparteine, ʟ-anagyrine) (León-González et al., 2018; Nur-e-Alam et al., 2019; Elsabrouty et al., 2024). Although the extensive research on *R. raetam* pharmacological properties, little is known about its burn wound healing effects.

Therefore, this study aimed to (*i*) evaluate *in vivo* the antioxidant, antimicrobial, and the potential of *R. raetam* leaves aqueous extract to treat burned skins in rat, and to (*ii*) decipher the mechanism underlying the possible wound healing property. An *in silico* study of the anti-inflammatory activities of compounds identified in the aqueous extract were also performed.

## 2 Materials and methods

### 2.1 Chemicals

Tris base (hydroxymethyl) aminomethane (20 mM), sodium chloride (NaCl), trichloroacetic acid (TCA), butylated hydroxytoluene (BHT), chlorohydric acid (HCl), 2-thiobarbituric acid (TBA), sodium carbonate (Na_2_CO_3_), copper sulfate (CuSO_4_), potassium sodium tartrate tetrahydrate (KNaC_4_H_4_O_6_,4H_2_O), Formalin, Folin–Ciocalteu reagent, Mueller Hinton (MH) agar and standards (cathechin, naringenin, quercetin, kaempferol, myricetin, amentoflavone, and rosmarinic, furilic, caffeic, and syringic acids) for phenolic quantification were purchased from Sigma-Aldrich (St. Louis, MO, United States). Methanol of HPLC grade was purchased from Carlo Erba Reactive-CDS (Val de Reuil, France). Biafine emulsion cream for burn treatment was bought from a local pharmacy.

### 2.2 Plant material and preparation of aqueous extract

Leaves of *R. raetam* (Forssk) Webb & Berthel were collected at the flowering stage in the region of Gafsa (Southwestern Tunisia, Latitude: 34°.25′59” (E), 8°.46’.07” (N)). This plant was identified by Pr. Mohamed Chaieb, botanist at the University of Science of Sfax (Tunisia). The voucher specimens of *R. raetam* (RE 207) are deposited at the Herbarium of the Faculty of Pharmacy of Monastir. Leaves were air-dried in the shade at ambient temperature (25°C ± 3°C) and then grounded, and subsequently used for the preparation of aqueous extract. To this end, leaf powder was macerated in bidistilled water at a ratio of 1:20 (w/v) for 24 h. The macerate was filtered through Wattman filter paper, frozen and lyophilized in a Christ-Alpha 2-4 freeze drier (Osterode, Germany). The resulting aqueous extract was stored at −20°C in airtight and amber flasks until use.

### 2.3 Characterization of the aqueous extract

The identification of phenolic compounds in leaf aqueous extract of *R. raetam* was performed using HPLC system following the method described by Athmouni et al. (2019). The system used was an Agilent 1,260 infinity II (Agilent technologies, Santa Clara, CA, United States) equipped with a diode array detector (DAD) and (a Zorbax Eclipse XDB) C18 column (4.6 × 100 mm, 3.5 μm particle size) maintained at 25°C. The injected sample volume was 20 μL. The separation was achieved using the mobile phases consisted of 0.1% formic acid in milli-Q water (solvent A) and 0.1% formic acid in methanol (solvent B) with a flow rate of 0.4 mL/min and the following gradient elution program: 0–5 min, 10%–20% A; 5–10 min, 20%–30% A; 10–15 min, 30%–50% A; 15–20 min, 50%–70% A; 20–25 min, 70%–90% A; 25–30 min, 90%–50% A; 30–35 min, return to initial conditions. The identification of eluted compounds based on their UV-vis. spectra recorded in the range of 200–400 nm, and by comparison of their retention times (RT) with those of reference standards. For the quantitative analysis, a calibration curve was obtained by plotting the peak area against different concentrations for each identified compound at 280 nm.

### 2.4 *In vivo* burned skin healing assay

#### 2.4.1 Animals and induction of burns

Twenty wistar rats were housed under 12 h light/dark cycles, temperature of 22°C ± 2°C and 40%–45% humidity for 2 weeks. They were fed balanced food pellets purchased from the (Société de Nutrition Animal (SNA), Sfax, Tunisia) and tap water, free *ad libitum*. For the induction of burns, animals were anesthetized using a single intraperitoneal of pentobarbital (40 mg/kg). The hairs of the dorsal lumbar part were carefully shaved, and deep third degree burns were generated using 22 mm diameter copper piece heated at 100°C for 3 s.

All animal experimentations were approved by animal Ethics Committee of Gafsa University (G/A/SV/2016/001) and carried out in accordance of the European Directive on Protection of Animals Used for Scientific Purposes (2010/63/EU).

#### 2.4.2 Experimental design

The animals were distributed randomly into four groups of five rats each. Group 1 (Br): rats burned and untreated (positive control); Group 2 (Br + S): rats burned and treated topically with biafine cream; Group 3 (Br + P): rats burned and treated topically with *R. raetam* aqueous extract and; Group 4 (T): rats without burn (negative control). Groups 2 and 3 were treated daily for 17 days.

At the end of the experimental period, animals were euthanized by cervical dislocation, and samples of burned skin parts of each group of rats were carefully removed and used for the estimation of the level of bacterial infection, determination of oxidative stress parameters and for histopathology examination.

#### 2.4.3 Measurement of the scaring

The surface of the wound was reproduced on a transparent paper and measured (mm^2^) every 3 days until full healing. The degree of its contraction reported as the percentage closure of the initial wound area was calculated as follows (Pitafi et al., 2024):

$$Contraction\;\left(\%\right)=\frac{\left(S0-Sn\right)}{S0}\times 100$$

Where S_0_ and S_n_ designate the area of the wound on day 0 and on day n^th^, respectively.

#### 2.4.4 Histopathology

The histological evaluation was performed at the end of the experiment on D17 after sacrifice of all the animals. The biopsies were carried out by taking a sample from the scar using a scalpel. Fragments of wound skin collected from different experimental groups of rats were sectioned and immediately fixed in a 4% formaldehyde solution for a minimum 48 h. Tissues are dehydrated in ethanolic solutions with an increasing concentration gradient (from 70% to 100%), followed by clearing with xylene. Thereafter, tissues were embedded in paraffin wax, cut into 4 µm thick sections, and mounted on slides for microscopy. Then slices were dewaxed, cleaned, and stained with haematoxylin and eosin (H & E) before being observed under a light microscope. This procedure is useful to visualize tissue structures.

#### 2.4.5 Antimicrobial activity

An amount of 50 mg of skin tissues from different groups were divided into small pieces and homogenized in 2 mL of a 0.9% *sodium chloride* (NaCl) physiological solution. Each homogenate was poured on petri dishes containing an agar-agar complete culture medium and incubated for 24 h at 37°C. Then the density of the formed colonies was estimated using semi-automatic colony counter device.

#### 2.4.6 Oxidative stress parameters

To evaluate the oxidative stress parameters in the injured skin, 300 mg of fresh fragment was collected from the skin tissue of different groups of rats, and homogenized in phosphate buffer saline (KH2PO4/K2HPO4) at pH 7, and centrifuged at 9,000 × g for 15 min at 4°C. The resulting supernatants were then used for the estimation of the lipid peroxidation and the activity of antioxidant enzymes.

The extent of lipid peroxidation estimated in term of malondialdehyde (MDA) was determined calorimetrically using the thiobarbituric acid reactive substances (TBARS) following the method of Buege et Aust (1978). Briefly, 0.1 mL of tissues supernatant was mixed with 2 mL of TBA-trichloroacetic acid-HCl reagent (0.37% TBA, 0.25 M HCl, 15% TCA; ratio 1:1:1) and incubated at 90°C for 15 min. After cooling, the obtained solution was centrifuged at 3,500 × *g* for 10 min, and the absorbance of the reaction mixture was measured at 535 nm and the MDA content was determined using the extinction coefficient of 1.56 × 10 M^-1^ cm^−1^.

For the activity of superoxide dismutase (SOD), the method of Misra (1972) based on the inhibition of radical-mediated chain propagating autoxidation of epinephrine in alkaline pH at 480 nm. The SOD activity was expressed as UI/mg of proteins.

For catalase activity (CAT) activity, the method of Aebi (1984) based on H_2_O_2_ decomposition at 240 nm was used. The reaction mixture contained 1 mL of 10^–2^ M phosphate buffer (pH 7), 0.1 mL of tissue supernatant and 0.4 mL of H_2_O_2_. The reaction was stopped by the addition of 2 mL of dichromate-acetic acid reagent and the CAT activity was determined using the coefficient of extinction of 40 mM and expressed as mM of H_2_O_2_ consumed min^–1^ mg^–1^ of proteins.

The GPx activity was estimated following the method of Flohé and Günzler (1984). Briefly, the reaction mixture contained 0.2 mL of 0.4 M phosphate buffer (pH 7), 0.1 mL of 10 mM sodium azide, 0.2 mL of tissue supernatant, 0.2 mL of GSH, and 0.1 mL of 0.2 mM H_2_O_2_ was incubated at 37°C for 10 min. The reaction was stopped by adding 0.4 mL of 10% TCA, centrifuged at 1,500 × *g* and the absorbance was measured at 412 nm. The GPx activity was expressed as GSH UI min^–1^ mg^–1^ of protein. Protein concentration was determined using bovine serum albumin (BSA) as standard (Bradford, 1976).

### 2.5 Computational molecular docking

The major compounds identified in the aqueous extract of *R. raetam* leaves powder were investigated for their potential as ligands for the prominent inflammatory factors in burning physiopathology: tumor necrosis factor-α (TNF-α) and interleukin-1β (IL-1β) receptors. Their respective structure was downloaded from PubChem database (https://pubchem.ncbi.nlm.nih.gov/) Quercetin (CID: 5280343), Kaempferol (CID: 5280863), Naringenin (CID: 439246), Myricetin (CID: 5281672), and Caffeic Acid (CID: 689043). The structure of Biafine (triethanolamine) was obtained from the Drug Bank database (https://go.drugbank.com/). These molecules were converted into 3D structures using the Corina server (https://demos.mn-am.com/corina.html), transformed into PDB format using BIOVIA Discovery Studio 2017 (https://discover.3ds.com/discovery-studio-visualizer-download), then reconverted into a dockable PDBQT format using Autodock tools. The crystal structures of TNF-α receptor (PDB ID: 3L9J) and IL-1 receptor (5BVP) were downloaded from the Protein Data Bank (PDB). Autodock Vina (version 1.5.7) (https://ccsb.scripps.edu/mgltools/). that was used to perform molecular docking studies. The ligand binding site was positioned at the centre of the grid box. A configuration file was created based on the dimensions of X = 33.496, Y = 60.57, Z = 16.642 for TNF-α receptor and X = 6.19, Y = 1.558, Z = −29.357 for IL-1β receptor, as determined by Discovery Studio’s visualizer (https://discover.3ds.com/discovery-studio-visualizer-download/). The analysis of binding interactions between the best-docked ligands and receptors was visualized using BIOVIA Discovery Studio (2017 R2) visualizer (https://discover.3ds.com/discovery-studio-visualizer/). The toxicity (carcinogenicity, mutagenicity, developmental toxicity and skin irritation and sensitization) of the plant phytochemicals and products contained in the cream Biafine was investigated using VEGA software 1.1.5 and QSAR approaches (https://www.vegahub.eu/download/vega-qsar-download-versions/).

### 2.6 Statistical analysis

All experiments were performed in triplicate and the results were expressed as means ± standard deviation (*n* = 3). One way of variance (ANOVA) followed by Tukey’s *post hoc* test for multiple comparisons was used for the inter-group comparison at the significance level of *p* < 0.05. All statistical analyses were carried out using Graph Pad Prism (GraphPad Software, San Diego, California, United States).

## 3 Results and discussion

### 3.1 Major phenolic compounds

**FIGURE 1:**
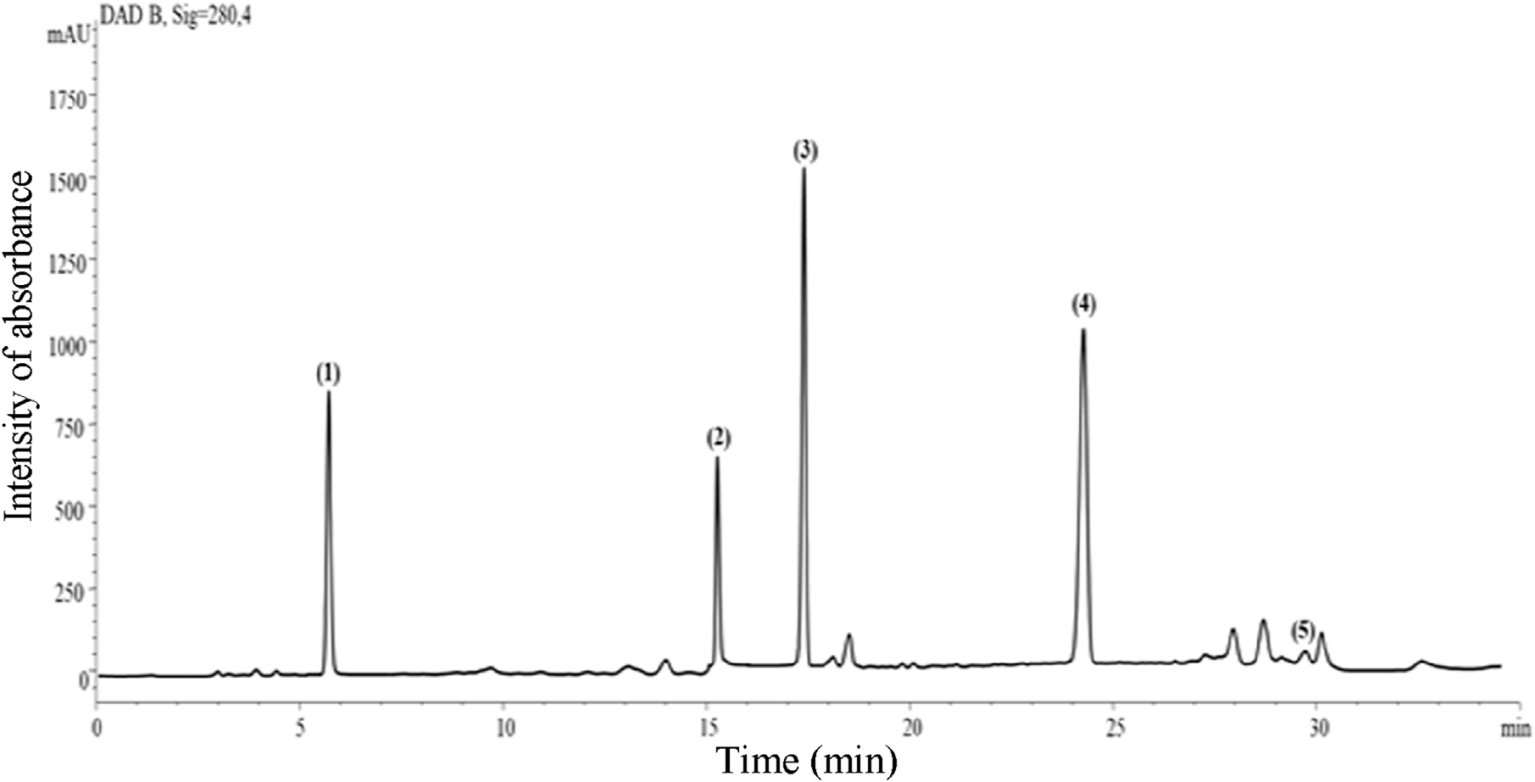
Representative HPLC-DAD chromatogram (acquisition at λ 280 nm) of *R. raetam* leaves aqueous extract. Peak numbers corresponded to (1) quercetin, (2) kaempferol, (3) caffeic acid, (4) naringenin, and (5) myricetin.

The identification of the main phenolic compounds in the aqueous extract of *R. raetam* was based on the comparison of their retention time and UV-vis. spectra with those of authentic standards (Figure 1). They included the flavonols quercetin (2.04 mg/g extract), kaempferol (1.74 mg/g extract), and myricetin (0.34 mg/g extract), the flavanone naringenin (2.32 mg/g extract) and the phenolic acid caffeic acid (3.56 mg/g extract). The presence of kaempferol, caffeic acid, quercetin and myricetin has already been reported in the Algerian (Djeddi et al., 2013), Tunisian and Saudi samples of *R. raetam* (Saada et al., 2014; Krayem et al., 2025).

### 3.2 Effect of *R. raetam* extract on microbial infection

**FIGURE 2:**
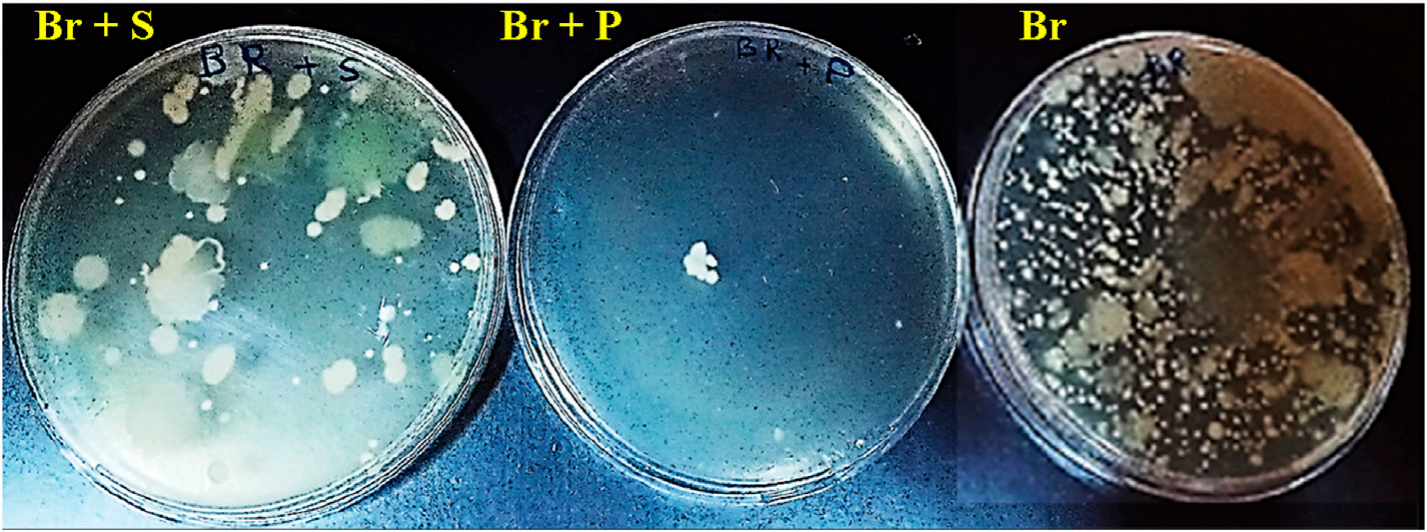
Images of the petri dishes showing the antimicrobial activities of *R. raetam* leaves extract and the biafine cream on spontaneous development of microbial colonies contained in burnt skin tissue homogenates of the respective treated rats (Br + P) and rats (Br + S) in comparison with that of untreated rats as a control (Br).

The topical application of *R. raetam* extract resulted in 99.68% reduction of spontaneously formed microbial colonies in the burnt tissues. The cleaning potential of the extract surpassed that of the standard biafine (98.19%) (Figure 2). The excellent antibiotic effect of *R. raetam* product could be beneficial for the wound healing process. The broad-spectrum antimicrobial activity of the identified components such as quercetin (Majumdar and Mandal, 2024), kaempferol (Periferakis et al., 2022), myricetin (Taheri et al., 2020), caffeic acid (Khan et al., 2021) and naringenin (Cai et al., 2023) could explain the observed antibiotic effect of *R. raetam* extract. The individual or joint effects of the identified compounds on bacterial growth inhibition in wound skin were presumably mediated through membrane disruption and or/depolarization, inhibition of bacterial virulence, inhibition of bacterial envelope synthesis, perturbation of lipid metabolism, inhibition of nucleic acid synthesis, reduction of respiratory activity and energy metabolism, inhibition of efflux pumps, inhibition of fungal mycelial growth, and inhibition of viral replication (Taheri et al., 2020; Nguyen and Battacharya, 2022; Veiko et al., 2023).

### 3.3 Effect of *R*. *raetam* extract on wound healing

**FIGURE 3:**
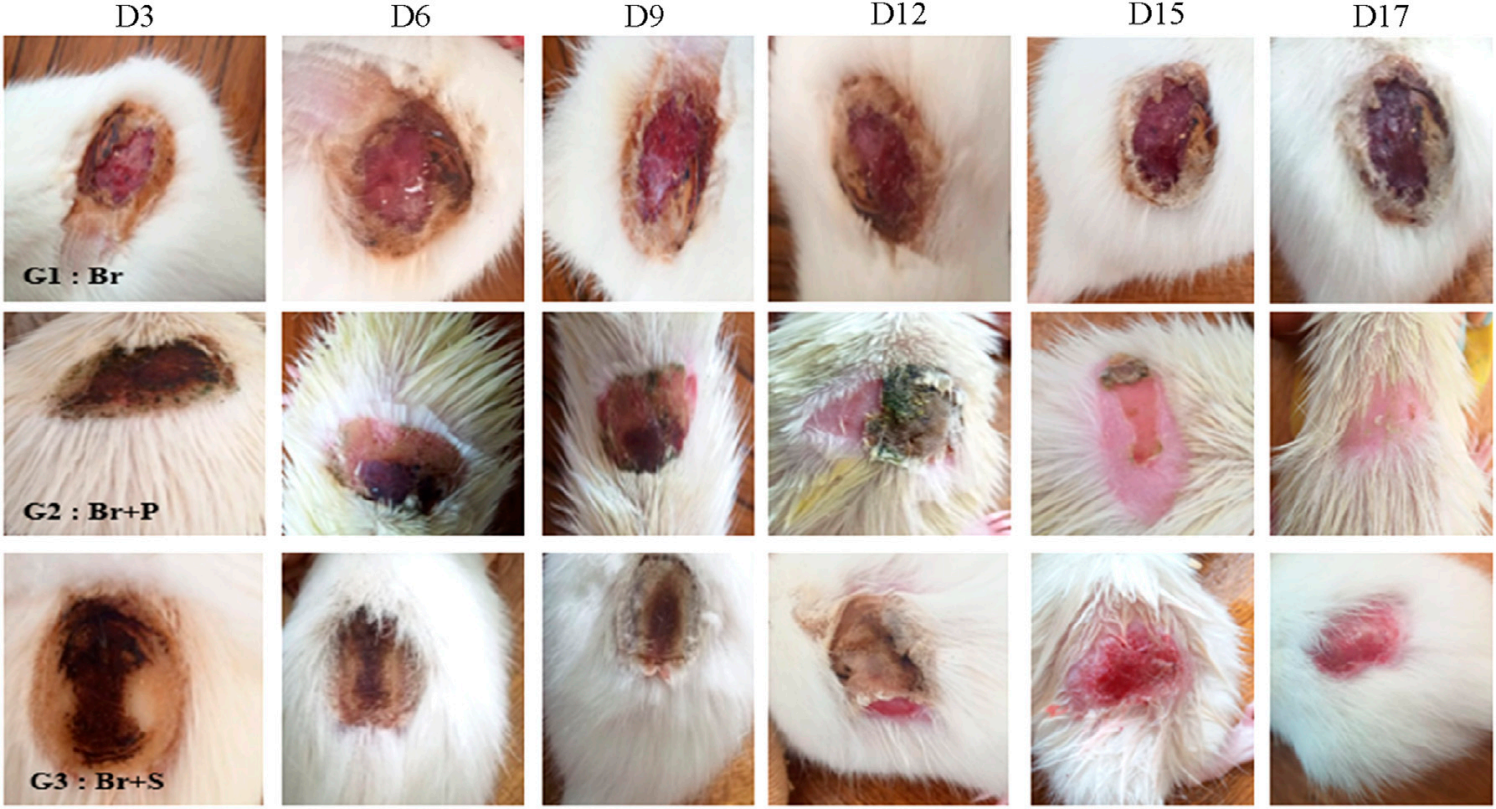
Evolution during the time of the skin burn healing in different groups of rats G1: untreated (Br), G2: treated with *R. raetam* leaves extract (Br + P), and G3: treated with conventional biafine cream (Br + S).

**FIGURE 4:**
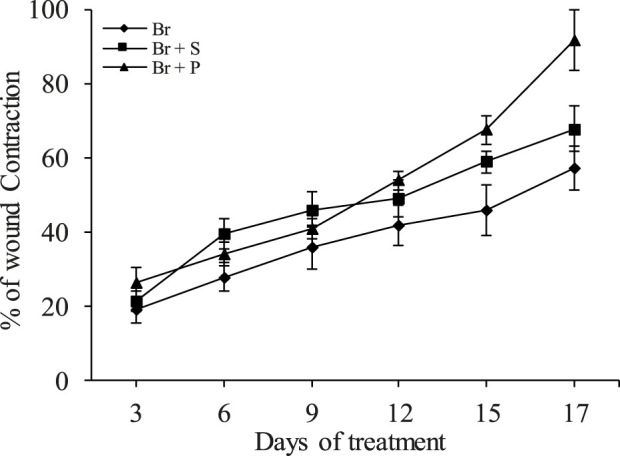
Percentage of wound contraction in rats of untreated group (Br), group treated with *R. raetam* leaves extract (Br + P), and group treated with conventional cream (Br + S). On the different curves the values represent the measurement carried out for three representative rats ±standard deviation.

The evolution of the scarring in different groups is presented in (Figure 3). Macroscopic examination showed a complete wound closure and disappearance of necrosis in wound skins of animals treated with *R. raetam* aqueous extract and the cream biafine. Quantitative evaluation based on percentage of contraction during the experimental period revealed a superior healing property of *R. raetam* aqueous extract over the cream biafine (Figure 4). The measurement of wounds width every 3 days (from day 3 to day 17) during the period of treatment, allowing the calculation of their surface and the determination of their percentage of contraction, proved that *R. raetam* leaves’ powder was more efficient and faster than conventional pharmaceutic cream in wound-healing process. After 17 days of the topical application of treatments, the wound contraction percentage in rat (Br + P) treated with the plant extract (91.3 ± 8.7%) was significantly higher (p ≤ 0.05) than in those (Br + S) receiving the pharmaceutic cream (70.2 ± 7.2%) and the control (Br) group (57.4 ± 8.3%). On the 17th day, at the end of treatment, the difference between the group treated with the cream and the group treated with the plant product is clearly significant.

### 3.4 Histological analysis

**FIGURE 5:**
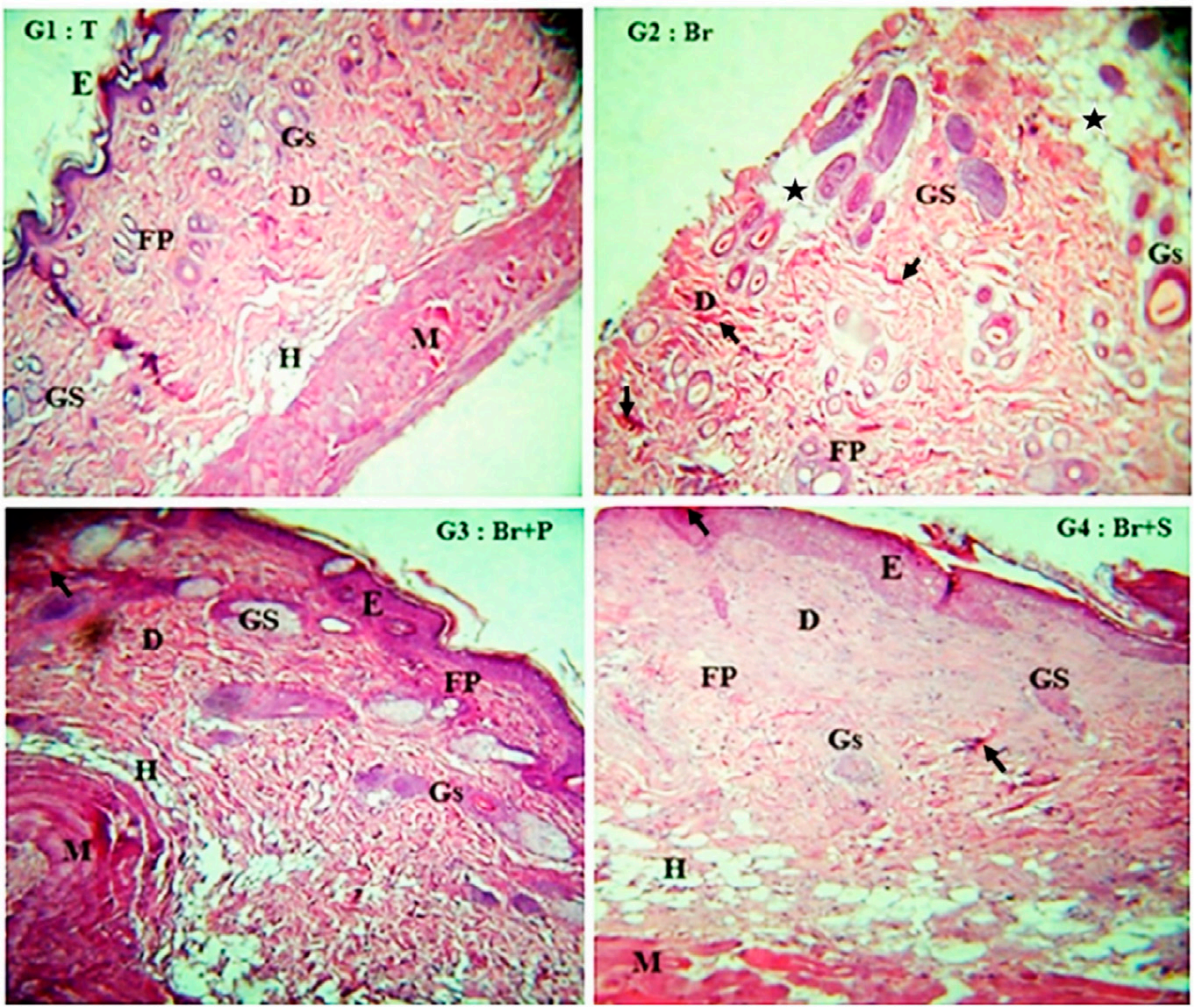
Microscopic observation (×100) of the histological sections of the necrotic zone of burnt skin tissue from the different groups of rats, untreated rats (Br), rats treated with *R. raetam* leaves extract (Br + P), rats treated with biafine cream (Br + S), and skin tissue of healthy rats (T). Designation of letters in images, E, epidermis; D, dermis; H, hypodermis; GS, sebaceous gland; Gs, sweat gland; FP, hair follicle; M, muscle.

In Figure 5, microscopic observation of the H & E stained sections showed that the skin tissue of control group of healthy rats has a structured and well organized part (epidermis, dermis and hypodermis). Cells of the epidermis and dermis layers were thermally destroyed on the skin of burnt rats (Br). These alterations triggered an inflammation and an installation of a large necrosis and hemorragic coagulation in the dermis as showed by asterix and arrows. Treatment of skin tissue of rat from (Br + P) group with the *R*. *reatam* leaves product triggered histological adjustments with inside the skin tissue as compared to the burnt rats (Br) group. Treatment with *R. raetam* product appeared renewing the epithelium and restoring the skin’s structure. The dermis confirmed advanced thickness and cell organization, with a well stratified squamous keratinized epithelium. Such healing property was associated with a well arranged dermal collagen fibers, a minimal residual edema a shortage of inflammatory cellular infiltration. Hair follicles and sebaceous glands seemed regular in shape and distribution, suggesting advanced tissue regeneration. The hypodermis was obviously with normal features. It should be noted that despite the satisfactory results, wound skin treated with biafin cream showed a slight interstitial edema in the dermis with torturous and less organized collagen fibers. Furthermore, the papillary dermal region, beneath the epithelium appears less thick in comparison to healthy group of rats (T). Overall, the potential healing properties of *R. raetam* might be linked to the antimicrobial and anti-inflammatory effects of its bioactive components. Naringenin (Salehi et al., 2019), quercetin (Ahmed et al., 2018; Aghababaei and Hadidi, 2023), caffeic acid (Song et al., 2008), kaempferol (Özay et al., 2019) and myricetin (Elshamy et al., 2020) were acknowledge by their anti-inflammatory actions *via* reducing leukocytes recruitment and downregulation of proinflammatory mediators/pathways TNF-α, NF-кβ, IL-6, IL-1β, NO, PGE2, caspase-3 and LTB4. Additional mechanisms involving antioxidant capacity (i.e., scavenging free radicals, reducing lipid peroxidation, and activation of antioxidant enzymes), stimulation of collagen fibrils formation and deposition, activation of the expression of vascular endothelial growth factor (VEGF) and fibroblast growth factor (FGF) *via* the Wnt/β-catenin signaling pathway, stimulation of neovascularization, improvement of cellular re-epithelialization and cell proliferation, have been also described in the wound healing process (Trinh et al., 2022; Riaz et al., 2024).

### 3.5 Effect of *R. raetam* extract on oxidative stress parameters

The possible implication of an antioxidant mechanism behind the manifest wound healing property of *R. raetam* extract was studied. In comparison with the untreated and healthy control, skin burning resulted in 2.5, 2.3, and 1.6-fold increase of the MDA content in burned and untreated rats, burned animals treated with biafin cream and *R. raetam* extract, respectively (Table 1). This indicates that burned animal experienced severe oxidative stress as reflected in the extended lipid peroxidation. This is consistent with the earlier studies linking wounds with oxidative stress and increased MDA levels (Schäfer and Werner, 2008). The state of oxidative stress was further exacerbated with the depletion of antioxidant enzymes SOD, CAT and GPx (Table 1). Treatment with *R. raetam* extract and to lesser extent the biafine cream restored approximately the normal oxidative state as revealed by the reduced MDA level and increased activity of SOD, CAT and GPx, alleviating thus oxidative stress in the wound and speed up its healing.

**TABLE 1:** Variation in the level of activities of lipid peroxidation (by Malondialdehyde (MDA) assay), superoxide dismutase (SOD), catalase (CAT) and glutathione peroxidase (GPx) in the homogenates of burnt skin tissues taken from different groups of rats, untreated rats (Br), rats treated with *R. raetam* leaves product (Br + P), rats treated with biafine cream (Br + S), and healthy rats (T).

Cellular antioxidant enzymes	T	Br	Br + P	Br + S
Different groups of rats				
Malondialdehyde (MDA)	0.31 ± 0.04	0.78 ± 0.08^###^	0.49 ± 0.15**	0.72 ± 0.16**
Superoxide dismutase (SOD)	5.11 ± 0.38	1.70 ± 0.18^###^	5.04 ± 0.66***	5.10 ± 0.55***
Catalase (CAT)	0.76 ± 0.16	0.12 ± 0.07^###^	0.49 ± 0.15*	0.72 ± 0.16***
Glutathione peroxidase (GPx)	2.25 ± 0.37	0.24 ± 0.09^###^	2.55 ± 0.46***	2.38 ± 0.42***

^###^: p ≤ 0.001 highly significant compared to Healthy rats.

Abbreviation: MDA, malondialdehyde; SOD, suerocide dismutase; CAT, catalase; GPx, Glutathione peroxidase. *p ≤ 0.05 significant compared to untreated Burnt rats; **p ≤ 0.01 moderately significant compared to untreated Burnt rats; ***p ≤ 0.001 highly significant compared to untreated Burnt rats.

The inhibitory effect of *R. raetam* extract on lipid peroxidation was unequivocally attributed to its phenolic composition, namely, quercetin, naringenin and kaempferol recognized for their potential free radical scavenging capacity and stimulatory effect on endogenous enzymatic and non-enzymatic antioxidant enzymes (Zandi and Schnug, 2022; Zulkefli et al., 2023). An enhanced migration of skin keratinocytes and fibroblast, cell-cell and cell-matrix interactions, and collagen synthesis have also been reported in the presence of antioxidants in successful healing process (Beserra et al., 2018; Phiboonchaiyanan et al., 2024). The observed wound-repairing property of *R. raetam* leaves extract might be primarily attributed to its strong antioxidant and antimicrobial activities.

### 3.6 *In silico* study

#### 3.6.1 Computed modelling of bioactive phenolics interactions with TNF-α and IL-1β receptors

**FIGURE 6:**
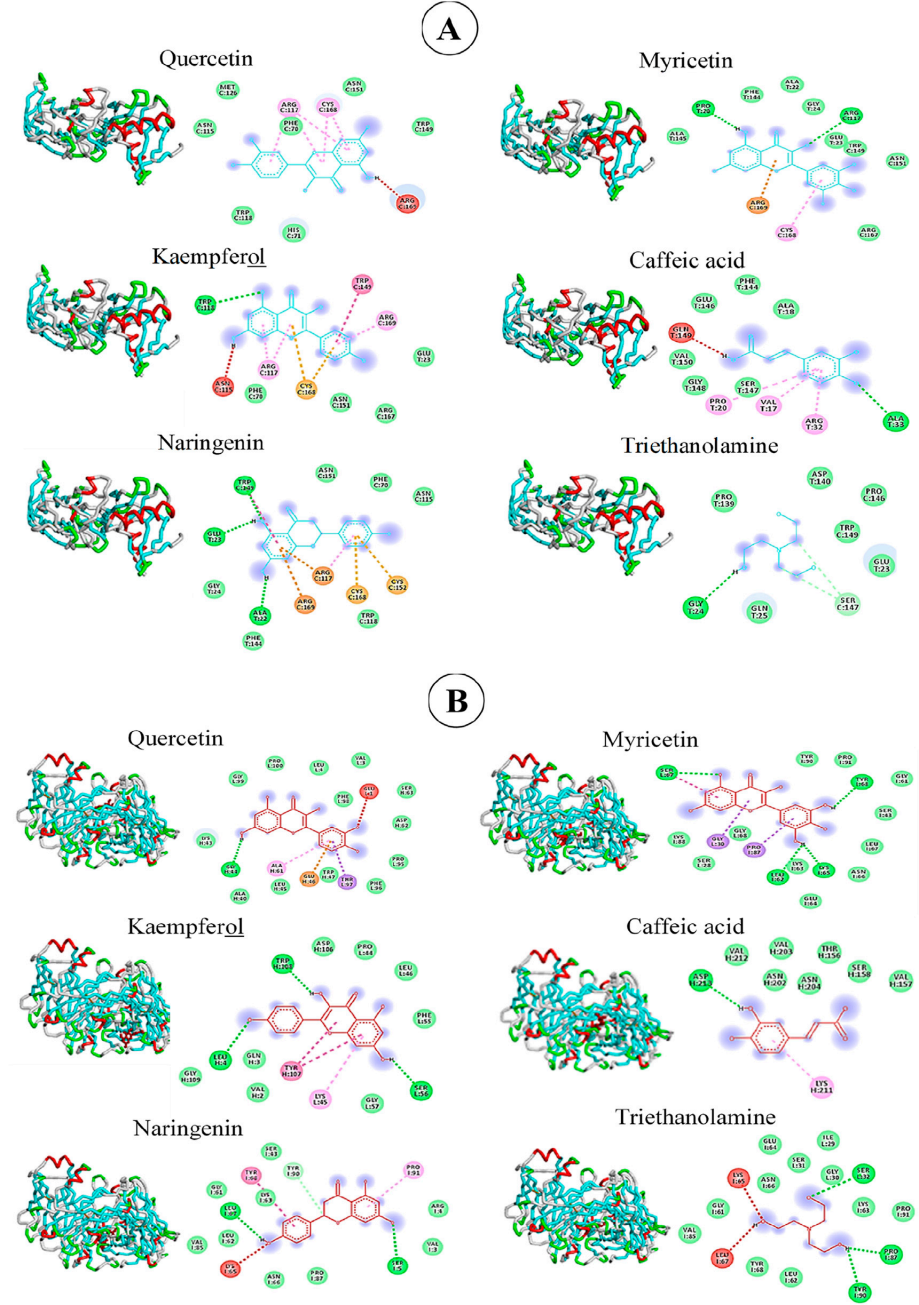
Interactions of quercetin, myricetin, kaempferol, naringenin, caffeic acid and triethanolamine with TNF-α-receptor **(A)** and IL-1β-receptor **(B)**.

The computed modelling of the interactions between *R. raetam* phenolics and receptors of pro-inflammatory factors showed in Table 2 that naringenin exhibited the highest biding affinity (−7.3 kcal/mol) to TNF-α receptor and forms three covalent hydrogen links with 3 amino acids (ALA 22, GLU, 23 and TRP 149) of the core of the site (Figure 6A). However, Quercetin, kaempferol, myricetin and caffeic acid showed one or two hydrogen covalent links to the receptor with a binding energy varying from −5.9 to −6.7 kcal/mol. Products-contained biafine cream established a unique hydrogen bond with the GLY024 residue at the site of TNF-α-R. The Van der Waals calculated energy revealed that naringenin present stable interaction with 6 amino acid residues at the site of TNF-α receptor suggesting its good fitting to dock into TNFα receptor site. Myricetin and quercetin have lower covalent binding to TNFα receptor (2 and 0 amino acids, respectively), but exhibited important allosteric interaction to the site with predicted Van der Waals links with eight and seven residues, respectively. Kaempferol, myricetin and naringenin bounded also non-covalently to ARG 117 and 169 and CYS 152 and 168 through sulphur interactions, thus providing good docking to the site of the TNF-α receptor (Figure 6A).

**TABLE 2:** Interaction scores of quercetin, myricetin, kaempferol, naringenin, caffeic acid and the standard triethanolamine with TNF-α and IL-1β receptors.

Identified compounds	Kcal/mol	H-bounds	Interaction of amino acids	Van der waals
Interaction parameters				
TNFα-R				
Quercetin	−6.7	0		7
Kaempferol	−6.7	1	TRP118	4
Naringenin	−7.3	3	ALA022, GLU023, TRP149	6
Myricetin	−6.7	2	PRO020, ARG117	8
Caffeic acid	−5.9	1	ALA033	6
Triethanolamine	−3.2	1	GLY024	6
IL-1β-R				
Quercetin	−7.2	1	GLY044	13
Kaempferol	−6.9	3	LEU4, SER56, TRP108	8
Naringenin	−7.1	2	LEU067, SER005	10
Myricetin	−7.2	4	LEU062, LYS065, SER067, TYR068	11
Caffeic acid	−5.7	1	ASP 213	7
Triethanolamine	−4.6	3	SER032, PRO087, TYR090	11

The docking model, also, revealed that the studied molecules interact, at different degrees, with the IL-1β receptor (Figure 6B). Myricetin formed hydrogen-bond with four residues (LEU062, LYS065, SER067 and TYRO68) of the receptor with an energetic affinity of −7.2 kcal/mol which make it the best fitting molecule to this protein. Kaempferol and triethanolamine, however, established only three H-binding to the site domain of IL-1β receptor with a docking score, of −6.9 and −4.6 kcal/mol respectively. Based on the number of residues interacting with the studied molecules in an electrostatic stable manner and in accordance to the calculated Van der Waals energy, the following order can be established: Quercetin (13 aa) > myricetin (11 aa) = triethanolamine (11 aa) > naringenin (10 aa) > kaempferol (8 aa) > caffeic acid (7 aa) (Table 2). These results suggest that phenolics of *R. raetam* leaves interact differentially with TNF-α and IL-1β receptors. At this point, it seems that the wound healing properties of quercetin, naringenin, myricetin and kaempferol was mediated through their inhibitory effect on the interaction and expression of pro-inflammatory cytokines TNF-α and IL-1β (Cai et al., 2023). By analysing compound-target network of eight phenolic compounds with potential target proteins, Shady et al. (2022) showed that quercetin, kaempferol and caffeic acid showed the highest connectivity with TNF-α, IL-1β, IL-6, Prostaglandin-endoperoxide synthase 1 (PTGS1), Vascular endothelial growth factor A (VEGFA), Epidermal growth factor receptor (EGFR), Matrix metallopeptidase (MMP1), MMP2, MMP3, MMP9, Arachidonate-15 lipoxygenase (ALOX15) (ALOX5), protein kinase AKT1 and the chemokine ligand 2 (CCL2). Cyclooxygenase (Cox 2), IL-6, inducible NO, prostaglandin E2 (PGE2), caspase-3, leukotriene B4 (LTB4), cluster of differentiation 68 (CD68), and NF-кB has also been described as potential target for the anti-inflammatory action-mediated wound healing property of quercetin, kaempferol, naringenin, myricetin and caffeic acid (Salehi et al., 2019; Chen et al., 2020; Elshamy et al., 2020; Kant et al., 2020).

#### 3.6.2 Computed prediction of the biodistribution and toxicity of *R. raetam* bioactive phenolics

Table 3 summarized a set of pharmacological properties of *R. raetam* chemicals using VEGA. HUB software and QSAR (quantitative structure activity relationship) modelling approach. The triethanolamine has the best aqueous solubility (−0.145 logS) while naringenin presented the lowest one (−3.19 logS). All tests phenolics presented good human intestinal absorption (HIA ≥0.8) but they are unable to cross the brain blood barrier (BBB). Of the test bioactive phenolics, only naringenin and caffeic acid have the potential to permeate through the caco-2 owing probably to their high infusibility across the lipophilic biomembrane.

**TABLE 3:** Biodistribution and Toxicity predictions of quercetin, myricetin, kaempferol, naringenin, caffeic acid and triethanolamine from *R. raetam* leaves.

Biodistribution and toxicity profile	Quercetin	Kaempferol	Naringenin	Myricetin	Caffeic acid	Triethanolamine
Biodistribution’ parameters						
Collision-induced dissociation (CID)	5280343	5280863	439246	5281672	689043	7618
Molecular weight (g/mol)	302.23	286.24	272.25	318.24	180.16	149.19
Water solubility (logS)	(−) 2.999	(−) 3.142	(−) 3.19	(−) 2.999	(−) 1.694	(−) 0.145
Rotable bonds	1	1	1	1	2	6
Cell line (Caco-2)	(−) 0.6417	(−) 0.8637	(+) 0.5424	(−) 0.7367	(+) 0.5000	(+) 0.7380
Brain blood barrier (BBB)	(−) 0.7750	(−) 0.8250	(−) 0.7750	(−) 0.7750	(−) 0.6500	(+) 0.5750
Human intestinal absorption (HIA)	(+) 0.9071	(+) 0.9499	(+) 0.9450	(+) 0.9071	(+) 0.9739	(+) 0.6710
Toxicity features						
Mutagenicity	(+) 1.0	(+) 1.0	(−) 0.0	(+) 0.45	(−) 1.0	(−) 1.0
Carcinogenicity	(−) 0.693	(−) 0.693	(+) 0.776	(−) 0.735	(−) 0.639	(−) 0.871
Acute toxicity	1,596.7	442.89	813.5	1,696.64	5121.68	5544.51
Skin sensitization	(+) 0.54	(+) 0.63	(+) 0.79	(+) 0.79	(+) 1.0	(−) 0.65
Skin irritation	(−) 0.729	(−) 0.745	(+) 0.762	(+) 0.716	(+) 0.818	(−) 0.842
Hepatotoxicity	(+) 0.844	(+) 0.844	(+) 0.856	(+) 0.81	Unknown	Unknown
Glucocorticoid receptor	Inactive	Inactive	Inactive	Inactive	Inactive	Inactive
Androgen receptor-mediated effect	Inactive	Inactive	Inactive	Inactive	Active	Inactive

Additional analyses showed that naringenin was neither mutagenic nor carcinogenic but it exhibited hight skin sensitivity, skin irritation and hepatoxicity scores, in addition to its relatively high acute toxicity. These attributes might limit its use as topical applicational for the wound healing. To overcame such problem, numerous approaches aimed at increasing its biodistribution and minimizing its skin toxicity could be applied. These include among others polymerization of cross-linked compounds, loading into carriers/nanocarriers such as nanoparticles, liposomes, or nanosuspensions, and its incorporation into active gels. It should be noted that caffeic acid and myricetin which have low acute toxicity without mutagenic and carcinogenic issues could also be considered as potential healing agents if issues linked to skin sensitivity and irritability were managed. To this end, the use of microemulsion/nanoemulsion, film, nanostructured lipid carriers, nanoparticles and ethosomes have been proposed as efficient tool to enhance their skin diffusion without side effects (Santos et al., 2019; Chutoprapat et al., 2022; Chauhan et al., 2025).

## 4 Conclusion

The wound healing effect of *R. raetam* leaves was evaluated for the first time. Leaf water extract particularly rich in quercetin, kaempferol, naringenin, myricetin and caffeic acid exhibited a manifest wound healing property and promote the skin regeneration. These effects were mediated through its (i) antibiotic effect on wound tissues, (ii) inhibitory effect on lipid peroxidation with a concomitant activation of endogenous antioxidant enzymes SOD, CAT and GPx, and (iii) the downregulation and inhibition of pro-inflammatory cytokine TNF-α and IL-1β interactions with ligands. The *in vivo* results were confirmed by *in silico* study where the putative healing bioactive phenolics naringenin and to a lesser extent kaempferol, myricetin, quercetin and caffeic acid showed strong interactions with TNF-α and IL-1β ligands. However, their application was hindered by their low solubility, toxicity, skin sensitivity and skin irritability. Enhancing their bioavailability, biodistribution and minimizing their toxicity warrant further studies. Additional in-depth studies aimed at investigating the mechanisms behind the wound healing property of individual and or/multiple phenolic bioactive compounds will be planned in the near future.

## Data Availability

The datasets presented in this study can be found in online repositories. The names of the repository/repositories and accession number(s) can be found in the article/supplementary material.
